# Intramuscular Fatty Acids in Meat Could Predict Enteric Methane Production by Fattening Lambs

**DOI:** 10.3390/ani11072053

**Published:** 2021-07-09

**Authors:** Francisco Requena Domenech, Pilar Gómez-Cortés, Silvia Martínez-Miró, Miguel Ángel de la Fuente, Fuensanta Hernández, Andrés Luis Martínez Marín

**Affiliations:** 1 Departamento de Biología Celular, Fisiología e Inmunología, Universidad de Córdoba, Ctra. Madrid-Cádiz km 396, 14071 Córdoba, Spain; v02redof@uco.es; 2 Instituto de Investigación en Ciencias de la Alimentación, Consejo Superior de Investigaciones Científicas (CSIC), Nicolás Cabrera 9, 28049 Madrid, Spain; mafl@if.csic.es; 3 Departamento de Producción Animal, Campus Mare Nostrum, Universidad de Murcia, 30100 Murcia, Spain; silviamm@um.es (S.M.-M.); nutri@um.es (F.H.); 4 Departamento de Producción Animal, Universidad de Córdoba, Ctra. Madrid-Cádiz km 396, 14071 Córdoba, Spain; pa1martm@uco.es

**Keywords:** fatty acids, meat, methane, lambs, vaccenic acid

## Abstract

**Simple Summary:**

The rearing of ruminant animals is an environmental issue related to greenhouse gas emissions, mainly methane. In the current study, a regression analysis was carried out to obtain a regression equation able to successfully estimate methane production of lambs fattened under intensive feeding systems from their meat fatty acid profile and average body weight during the fattening period. The optimized model exhibited high precision, accuracy and reproducibility. It showed a clear relationship between methane emissions and the fatty acid profile of lamb meat. Our predictive model indicates that fatty acid contents in intramuscular fat could be used to establish the environmental footprint of meat from ruminants.

**Abstract:**

Methane (CH_4_) emissions pose a serious problem for the environmental sustainability of ruminant production. The aim of the present study was to explore the usefulness of the intramuscular fatty acid (FA) profile to estimate CH_4_ production of lambs fattened under intensive feeding systems. A statistical regression analysis of intramuscular FA derived from ruminal metabolism was carried out to assess the best predictive model of CH_4_ production (g/d) in lambs fed with different diets. CH_4_ was calculated with three distinct equations based on organic matter digestibility (OMD) at maintenance feeding levels. The OMD of the experimental diets was determined in an in vivo digestibility trial by means of the indicator method. Regression models were obtained by stepwise regression analysis. The three optimized models showed high adjusted coefficients of determination (R^2^_adj_ = 0.74–0.93) and concordance correlation coefficients (CCC = 0.89–0.98), as well as small root mean square prediction errors (RMSPE = 0.29–0.40 g/d). The best single predictor was vaccenic acid (*trans*-11 C18:1), a bioactive FA that is formed in the rumen to a different extent depending on dietary composition. Based on our data and further published lamb research, we propose a novel regression model for CH_4_ production with excellent outcomes: CH_4_ (g/d) = −1.98 (±1.284)–0.87 (±0.231) × *trans*-11 C18:1 + 0.79 (±0.045) × BW (R^2^_adj_ = 0.97; RMSPE = 0.76 g/d; CCC = 0.98). In conclusion, these results indicate that specific intramuscular FA and average BW during fattening could be useful to predict CH_4_ production of lambs fed high concentrate diets.

## 1. Introduction

Nowadays, the environmental impact of the livestock sector in the form of greenhouse gas emissions (GHG), namely, nitrous oxide (N_2_O), carbon dioxide (CO_2_) and methane (CH_4_) is of great concern worldwide. Considering its global warming potential in terms of CO_2_ equivalents, CH_4_ represents 44% of the total GHG emissions of the livestock supply chain or 4% to 6.4% of the global anthropogenic GHG emissions [1,2]. Cattle and small ruminants contribute 65% and 6.5%, respectively, of GHG emissions in the livestock sector and enteric CH_4_ is about one-half of those emissions [1]. Thus, enteric CH_4_ emissions pose a serious problem for the environmental sustainability of ruminant production.

Enteric CH_4_ is a by-product of microbial fermentation of feed in the rumen and the hindgut of farm animals. Microbial digestion releases volatile fatty acids, CO_2_ and hydrogen (H_2_). Archaea methanogens produce CH_4_ from CO_2_ and H_2_, which have higher availability under feeding conditions that promote more active protozoa and fibrolytic bacteria populations [3]. In ruminants, it is estimated that around 2–12% of diet gross energy (GE) is lost in enteric CH_4_, depending largely on diet chemical composition and digestibility [4,5]. CH_4_ is mostly produced in the rumen and released into the atmosphere through the mouth and nostrils, with direct CH_4_ rectal emissions accounting for only 3% of the total [6].

As a result of such environmental concerns, many regression equations have been developed to predict enteric CH_4_ emissions from ruminant species, either in terms of CH_4_ yield (g/kg dry matter intake) or production (g or MJ/d) to evaluate different CH_4_ mitigation strategies. Most of the predictors used in these equations are based on animal or dietary composition [7,8,9,10,11,12], and also milk fatty acid (FA) profile in dairy ruminants [13,14,15,16], since there is an underlying relationship between diet and ruminal microbial population as well as an inherent association between ruminal lipid metabolism and milk FA profile [17]. Thus, it might be hypothesized that if milk FA profile can be related to CH_4_ emission in dairy ruminants, the intramuscular fat (IMF) FA profile of ruminants may also be related to CH_4_ production. However, to our knowledge, there is no available information on this subject.

In a recent paper, we showed that the intramuscular FA profile of meat could be a useful tool to monitor the feeding background of lambs entering the food supply chain [18]. The aim of the present study was to further explore the usefulness of the intramuscular FA profile to estimate the environmental impact of intensive lamb raising in terms of CH_4_ production.

## 2. Materials and Methods

This work was carried out with data from previous studies, the main results of which have been published elsewhere [19,20]. Briefly, a total of 105 intact male lambs of the Manchega breed with an initial bodyweight (BW) of 13.9 ± 1.7 kg and 35 ± 7 days old were randomly allocated to 15 straw-bedded pens at a commercial farm. Each pen had its own water and feed troughs and a straw rack. Animals had free access to water, concentrate feed and cereal straw during the whole experimental period. The pens were randomly allocated to one of three treatments (five replicates per treatment), which differed in the composition of the concentrate that was fed (Table 1): Control (CON), Camelina (CAM), and Fibrous (FIB). The concentrates were mixed and steam pelleted in a commercial feed mill (Iniciativas Alimentarias S.A., Ciudad Real, Spain). Diet composition was determined by usual procedures [21,22] and diet GE was calculated from chemical composition [11]. Average BW and concentrate intake per pen were recorded weekly for 6 weeks. Average daily dry matter intake (DMI, g/day) was calculated for each pen assuming that barley straw represented 5% of total DMI [23].

On day 42 of the trial, two lambs per pen (i.e., 10 lambs from each experimental treatment) with the final BW closest to the average pen BW, were tagged to track their carcasses. Then, all animals were sent to a commercial abattoir for slaughter. Samples of *Longissimus thoracis* muscle were obtained for intramuscular FA analysis after 6 days of aging at 4 °C. IMF was extracted following the Bligh and Dyer procedure [24] adding BHT as antioxidant, and total lipids were preserved in amber vials frozen at −17 °C until derivatization. Fatty acids were derivatized to methyl esters by base-catalyzed methanolysis and quantified by gas chromatography with an Agilent model 6890 N network system (Palo Alto, CA, USA) fitted with an autoinjector, a flame ionization detector (FID) and a CP-Sil 88 fused silica capillary column (100 m 0.25 mm i.d., Varian, Middelburg, The Netherlands), as described by Gómez-Cortés et al. [18,20]. A total of 59 FA were identified and quantified in lamb IMF. Eleven FA were selected for the statistical regression study (Table 2) based on their well-known relationship with ruminal bacteria metabolism [25,26].

CH_4_ production was calculated using three equations developed for ruminants in general: Equation (13) of Ramin and Huhtanen [11], the equation of Bell et al. [27] and Equation (4) of Sauvant et al. [28]. The predictors in these equations are feeding level or DMI relative to BW, organic matter digestibility (OMD) or digestible organic matter in dry matter (DOM), and the contents of ether extract, neutral detergent fibre and non-fibrous carbohydrates in dry matter basis. Since Equation (13) of Ramin and Huhtanen [11] reports CH_4_ as kJ per MJ of GE, the result was multiplied by GE intake in MJ/d and divided by the conversion factor 55.65 kJ/g to obtain CH_4_ production in g/d. GE intake was calculated from diet GE and DMI. DOM was calculated by multiplying OMD by organic matter in dry matter.

For stablishing OMD, we conducted a digestion assay at the Animal Nutrition Experimental Centre at the University of Murcia. A total of 15 rams (40.2 ± 2.3 kg and 7.8 ± 0.6 months old) kept in individual 1.5 m^2^ straw-bedded pens provided with individual feed and water throughs were used. Rams were randomly allocated to one of the three experimental treatments detailed above (5 replicates per treatment). Dietary concentrates (CON, CAM and FIB) and barley straw were offered ad libitum during 21 days. The adaptation period lasted 15 days and the collection period 6 days. Then, the ratio concentrate:straw was kept at 95:5 and the diet was offered at 90% of the intake registered the previous week to avoid refusals. Titanium dioxide (TiO_2_) was used as an inert marker [29]. Five grams of TiO_2_ per kg were added to the batch of each experimental concentrate in the mixer before pelletizing at the same commercial feed mill. Representative subsamples of each batch were collected, and aliquots were mixed to obtain a composite sample for each concentrate. Individual feces were sampled directly from the rectum, by hand protected by a single-use latex glove, on three alternate days of the 6-day collection period, just before morning feeding. Aliquots of the three subsamples from each lamb were mixed in a composite sample and kept in plastic zip bags that were stored at −20 °C until analysis. Organic matter in feed and feces was determined by the difference between the dry matter content after drying in an oven until constant weight and the ash concentration after incineration in a muffle furnace. Quantification of TiO_2_ in feeds and feces was done following the procedure of Myers et al. [30]. Then, OMD was calculated with the following formula: OMD (%) = [1 − (M_feed_/M_feces_)/(OM_feces_/OM_feed_] × 100; where M_feed_ and M_feces_ are the concentrations of TiO_2_ in feed and feces dry matter, and OM_feces_ and OM_feed_ are the concentrations of organic matter in feces and feed dry matter.

SAS University Edition 3.8 (SAS Institute, Cary, NC, USA) was used for the statistical analysis. The GLM procedure was used to investigate differences between dietary treatments in OMD, DMI relative to BW and calculated CH_4_ production. Experimental treatments were the fixed effect in the statistical model. When the model was significant, differences between least squared means were investigated with Tukey’s test. Stepwise regression analysis in the GLMSELECT procedure was used to investigate the relationship between calculated CH_4_ production and the contents of specific FA in lambs IMF. The best model was chosen according to the PRESS option (minimum true prediction error through leave-one-out validation). Model performance was assessed by the adjusted coefficient of determination (R^2^_adj_), the root of the mean square of prediction error (RMSPE), the RMSPE expressed as proportion of the observed mean (%RMSPE), and the concordance correlation coefficient (CCC). Furthermore, the mean square of prediction error (MSPE) was decomposed into mean bias (measure of precision), slope bias (measure of accuracy), and random error [12]. Pearson’s correlation was used when appropriate. Statistical significance was declared at *p* < 0.05.

## 3. Results and Discussion

The eleven FA selected for the statistical regression analysis (Table 2) were selected because they directly relate to rumen bacteria metabolism [25,26]. From a quantitative point of view, these FA are the most important of their respective groups, accounting for more than 60% of total FA within each group (i.e., linear odd, *iso* and *anteiso* FA groups, and *cis* and *trans* C18 isomer groups). Linear odd, *iso* and *anteiso* FA are synthesized by rumen bacteria and incorporated into their lipid membranes to maintain fluidity and cell membrane function [31]. On the other hand, *cis* and *trans* isomers of C18:1 and C18:2 FA are produced by bacteria in the ruminal biohydrogenation process from unsaturated lipids consumed in the diet [32]. Several FA included in the statistical analysis (C17:0 *iso*, *cis*-11 C18:1, *trans*-10 C18:1 and *trans*-11, *cis*-15 C18:2) have been previously related to CH_4_ production in dairy cows [13,14].

Lambs’ average BW during the fattening period was 19.8 ± 0.74 kg and did not differ between dietary treatments [19]. FIB concentrate exhibited lower OMD and higher feeding level (*p* < 0.05) when compared to CON and CAM rations. Accordingly, calculated CH_4_ production in the FIB group was lower than CON and CAM treatments (*p* < 0.05) (Table 3). It is worth mentioning that CH_4_ production calculated with the three equations was highly correlated (r = 0.90 to 0.98, *p* < 0.001). However, this similarity does not necessarily imply that the predictions of those equations are accurate. Moreover, it has been pointed out that equations to predict CH_4_ production in sheep need refinement [33], which increases the significance of our approach to predict methane production from meat FA profile.

Several equations have been developed to predict CH_4_ production specifically in sheep [9,10,34] or across ruminant species [11,27,28,35]. The three equations used in the present study (Table 3) fitted our data well. All of them rely on knowing OMD and present different levels of complexity according to the number of predictors used. The simplest model is Equation (4) of Sauvant et al. [28], which only takes into account DOM and feeding level. The equations of Ramin and Huhtanen [11] and Bell et al. [27] include feeding level and percentage of crude fat in the diet dry matter as predictors. The latter [27] also includes DOM at maintenance feeding level as a predictor whereas the former incorporates OMD at maintenance feeding level and the contents of neutral detergent fibre and non-fibrous carbohydrates in diet dry matter. Diet digestibility and feeding level play a pivotal role in the prediction of CH_4_ emissions by ruminants. However, the influence of carbohydrate and fat contents in the diet has been documented and should also be considered. Increasing diet digestibility, mainly through improved fibrous carbohydrate digestibility, increases CH_4_ emission, while increasing feeding level and dietary fat decrease it [35,36,37,38].

The best regression models obtained in the present study for predicting CH_4_ production from FA profiles, as shown in Table 2, are presented in Equations (1)–(3), respectively. In the statistical models to derive Equations (1)–(3), CH_4_ production was calculated according to Equation (13) of Ramin and Huhtanen [11], the equation of Bell et al. [27] and Equation (4) of Sauvant et al. [28], respectively.

CH_4_ (g/d) = 19.06 (±1.917) + 1.74 (±0.735) × C17:0–3.10 (±0.861) × *cis*-11 C18:1–1.07 (±0.260) × *trans*-11 C18:1 + 5.36 (±2.112) × *trans*-11, *cis*-15 C18:2 R^2^_adj_ = 0.74; RMSPE = 0.40 g/d; %RMSPE = 2.83%; CCC = 0.89 (1)

CH_4_ (g/d) = 24.50 (±2.282) + 2.43 (±0.695) × C17:0–11.09 (±2.462) × C14:0 *iso*–13.51 (±4.698) × C17:0 *iso*–2.62 (±0.726) × *cis*-11 C18:1–1.36 (±0.191) × *trans*-11 C18:1 R^2^_adj_ = 0.93; RMSPE = 0.34 g/d; %RMSPE = 2.28%; CCC = 0.98 (2)

CH_4_ (g/d) = 15.22 (±1.372)–11.95 (±3.425) × C17:0 *iso* + 4.18 (±1.165) × C17:0 *anteiso*–0.80 (±0.556) × *cis*-11 C18:1–1.03 (±0.138) × *trans*-11 C18:1 R^2^_adj_ = 0.91; RMSPE = 0.29 g/d; %RMSPE = 2.51%; CCC = 0.96 (3)

In all equations fatty acids are g/100 g of total fatty acids.

Equation (2) was slightly better than the others in terms of precision, accuracy and reproducibility, according to R^2^_adj_, RMSPE, %RMSPE and CCC values, respectively, but the three models performed very well. Moreover, their high precision and accuracy was supported by the fact that none of the models showed mean or linear bias, with nearly 100% of MSE due to random error. The best single predictor in Equations (1)–(3) was *trans*-11 C18:1 (R^2^ = 0.51, 0.78 and 0.78, respectively), whereas the weakest predictors were C17:0 (R^2^ improvement = 13.6%), C14:0 *iso* (R^2^ improvement = 4.2%) and *cis*-11 C18:1 (R^2^ improvement = 1.1%) in Equations (1)–(3), respectively. These results may be supported by the fact that previous studies in dairy cattle have found negative relationships between methane emissions and the contents of *cis*-11 C18:1, *trans*-11 C18:1 and C17:0 *iso* in milk fat, whereas C17:0 *anteiso* showed a positive relationship [13,14,39,40]. In contrast to our results, a negative relationship between *trans*-11, *cis*-15 C18:2 and methane emissions has been observed [13,14,39]. The role of C17:0 has been reported as positive with regard to CH_4_ yield and negative with regard to CH_4_ production [14,39,40].

Unfortunately, published papers on CH_4_ emissions of lambs that simultaneously report a detailed meat FA profile are scant. Therefore, in order to challenge our approach, we constructed a database with diet composition, average DMI and BW, and intramuscular FA profile of lambs from previous published works [41,42,43]. Then, we derived the OMD at maintenance levels in their diets from tabulated data of feed composition [44] and calculated CH_4_ production in two ways: (i) using Equation (4) of Sauvant et al. [28] (Table 4) and (ii) using intramuscular FA contents in Equation (3) of the present study, scaled to BW to include BW effects on DMI (Table 5). Equation (3) was chosen over Equation (2) because one publication did not report C14:0 *iso* contents in intramuscular fat [42]. It is important to note that [41,42,43] included about 6% of highly unsaturated oils in their studies, which is known to have a great impact on the intramuscular FA profile due to its effects on ruminal fermentation [45,46].

It was found that calculated CH_4_ with Equation (4) of Sauvant et al. [28] (Table 4) and Equation (3) of the present study (Table 5) were correlated (r = 0.52), although statistical significance was not reached (*p* = 0.10) due to the low calculated value of CH_4_ production with Equation (3) in the SH diet of Costa et al. [41]. Such a low value was derived from the high *trans*-11 C18:1 content in the SH treatment, which was three-fold higher than the average *trans*-11 C18:1 level in other treatments of that study (Table 5). Nevertheless, the results of the comparison supported the relationship between calculated CH_4_ production and intramuscular FA profile and encouraged us to develop a new and more generalizable regression equation including both our own data and those from Costa et al. [41], Oliveira et al. [42] and Santos-Silva et al. [43] (Table 2, Table 3, Table 4 and Table 5). In this new statistical model (Equation (4)), calculated CH_4_ production with Equation (4) of Sauvant et al. [28] was the variable, C17:0 *iso*, C17:0 *anteiso*, *cis*-11 18:1, *trans*-11 18:1 and average BW were the predictors, and the regression was weighted by the squared root of the number of observations in each experimental treatment.

CH_4_ (g/d) = −1.98 (±1.284)–0.87 (±0.231) × *trans*-11 C18:1 + 0.79 (±0.045) × body weight R^2^_adj_ = 0.97; RMSPE = 0.76 g/d; %RMSPE = 4.04%; CCC = 0.98 (4)

*Trans*-11 C18:1 is expressed in g/100 of total fatty acids and body weight is expressed in kg.

The intercept in Equation (4) did not reach statistical significance (*p* = 0.15) but both predictors were significant (*p* < 0.01). Our model showed high precision, accuracy and reproducibility, supported by excellent R^2^_adj_, RMSPE, %RMSPE and CCC values, and the fact that nearly 100% of MSE was due to random error. Equation (4) supports the important role of the ruminal biohydrogenation intermediate *trans*-11 C18:1 as a predictor of CH_4_, which would be related to the fact that *trans*-11 C18:1 is strongly related to the composition of the diet [20,41,43]. Although several assumptions had to be made in the construction of the model, the obtained results substantiated a general relationship between CH_4_ production and intramuscular FA profile in lambs, best explained taking into account the BW of the animals considered (Equation (4)). The effect of BW on CH_4_ production has been previously described in growing sheep [47].

In general, prediction models of CH_4_ production in ruminants are derived from studies where data were obtained with laboratory techniques that might not resemble what occurs under farming conditions [48]. The present model is even more limited since CH_4_ production was indirectly calculated and only data from lambs raised under intensive feeding conditions were used, which precludes its applicability to lambs fed in pastures or high forage systems. Clearly, there is a need for more studies that simultaneously report measures of CH_4_ production and intramuscular FA composition of lambs raised not only in feedlots but also in other feeding systems in order to develop more reliable and applicable predictive models of CH_4_ production.

## 4. Conclusions

The equations obtained in the present study might be potentially useful to account for the environmental impact of lambs raised under intensive farming conditions. These results support that specific intramuscular FA and average BW during fattening could be used to predict CH_4_ production in lambs. More studies would be needed to develop robust prediction models of CH_4_ production from intramuscular FA in lambs.

## Figures and Tables

**Table 1:** Chemical composition (%) and gross energy content (MJ/kg) of the experimental diets (values on dry matter basis).

Parameters	Treatments ^1^		
	CON	CAM	FIB
Ash	6.3	7.1	9.1
Crude protein	17.6	17.5	17.8
Crude fat	4.8	4.6	4.4
Neutral detergent fiber	18.2	20.5	36.8
Non-fibrous carbohydrates ^2^	53.9	51.1	32.8
Gross energy ^3^	18.6	18.5	18.4

^1^ CON: typical commercial concentrate rich in starch and based on cereals and soybean meal. CAM: typical commercial concentrate, which replaces 50% of soybean meal with camelina meal. FIB: concentrate rich in neutral detergent fiber, based on fibrous by-products and not including cereals or soybean meal. ^2^ Calculated as (100-ash-crude protein-crude fat-neutral detergent fiber). ^3^ Calculated as described by Ramin and Huhtanen [11].

**Table 2:** Mean (±standard deviation), minimum (Min) and maximum (Max) contents of fatty acids quantified in the *Longissimus* *thoracis* muscle of lambs fed the experimental diets that were selected to be included in the study. Data expressed as g per 100 g of total fatty acids.

Parameters	Treatments ^1^								
	CON			CAM			FIB		
	Mean	Min	Max	Mean	Min	Max	Mean	Min	Max
C15:0	0.34 ± 0.06	0.28	0.40	0.32 ± 0.01	0.31	0.33	0.24 ± 0.04	0.21	0.31
C17:0	1.45 ± 0.24	1.19	1.76	1.34 ± 0.06	1.24	1.39	0.84 ± 0.12	0.75	1.05
C14:0 *iso*	0.34 ± 0.05	0.28	0.39	0.24 ± 0.03	0.20	0.27	0.23 ± 0.04	0.18	0.28
C17:0 *iso*	0.14 ± 0.03	0.10	0.18	0.13 ± 0.02	0.10	0.15	0.14 ± 0.04	0.10	0.19
C17:0 *anteiso*	0.27 ± 0.05	0.21	0.33	0.45 ± 0.04	0.41	0.51	0.44 ± 0.07	0.36	0.56
*Cis*-11 C18:1	2.06 ± 0.22	1.79	2.25	2.14 ± 0.11	2.00	2.29	1.57 ± 0.11	1.47	1.76
*Trans*-10 C18:1	3.33 ± 1.22	1.20	4.21	4.77 ± 0.56	3.90	5.35	4.54 ± 0.98	3.60	5.81
*Trans*-11 C18:1	0.67 ± 0.06	0.59	0.76	1.26 ± 0.20	1.03	1.50	3.59 ± 0.62	3.25	4.70
*Trans*-11,*cis*-15 C18:2	0.04 ± 0.01	0.03	0.05	0.22 ± 0.02	0.21	0.25	0.27 ± 0.04	0.23	0.33
*Cis*-9,*trans*-11 18:2	0.18 ± 0.04	0.14	0.24	0.28 ± 0.05	0.24	0.35	0.79 ± 0.18	0.59	1.08
*Trans*-10,*cis*-12 C18:2	0.10 ± 0.01	0.09	0.11	0.10 ± 0.01	0.09	0.11	0.08 ± 0.00	0.08	0.08

^1^ CON: typical commercial concentrate rich in starch and based on cereals and soybean meal. CAM: typical commercial concentrate, which replaces 50% of soybean meal with camelina meal. FIB: concentrate rich in neutral detergent fiber based on fibrous by-products and not including cereals or soybean meal.

**Table 3:** Apparent in vivo organic matter digestibility (OMD, %) and digestible organic matter (DOM, % dry matter) of the diets at maintenance feeding level, and dry matter intake (DMI, kg/d), actual feeding level (g dry matter intake/kg body weight) and calculated methane (CH_4_) production (g/d) of lambs according to Ramin and Huhtanen [11], Bell et al. [27] and Sauvant et al. [28].

Parameters	Treatments ^1^			SEM ^2^	*p*
	CON	CAM	FIB		
OMD	76.7 ^a^	77.2 ^a^	66.4 ^b^	1.41	<0.001
DOM	71.8 ^a^	71.7 ^a^	60.4 ^b^	1.49	<0.001
DMI	0.80 ^b^	0.77 ^b^	0.96 ^a^	0.03	<0.001
DMI/kg BW	40.9 ^b^	38.2 ^b^	48.5 ^a^	1.31	<0.001
CH_4_ [11]	14.7 ^a^	14.5 ^a,b^	13.3 ^b^	0.24	<0.001
CH_4_ [27]	16.1 ^a^	16.0 ^a^	13.0 ^b^	0.43	<0.001
CH_4_ [28]	12.5 ^a^	12.4 ^a^	10.4 ^b^	0.30	<0.001

^1^ CON: typical commercial concentrate rich in starch and based on cereals and soybean meal. CAM: typical commercial concentrate, which replaces 50% of soybean meal with camelina meal. FIB: concentrate rich in neutral detergent fiber based on fibrous by-products and not including cereals or soybean meal. ^2^ Standard error of the mean. ^a,b^ Least squared means without a common superscript are significantly different by Tukey’s test at *p* < 0.05.

**Table 4:** Apparent organic matter digestibility (OMD, %) and digestible organic matter (DOM, % dry matter) of the diets at maintenance feeding level, body weight (BW, kg), dry matter intake (DMI, kg/d), actual feeding level (g dry matter intake/kg body weight), and calculated methane (CH_4_) production (g/d) of lambs allocated to several experimental treatments in the studies of Costa et al. [41], Oliveira et al. [42] and Santos-Silva et al. [43].

Treatments ^1^	OMD	DOM	BW	DMI	DMI/kg BW	CH_4_^2^
Costa et al. [41]						
CON	77.7	71.6	33.4	1.18	35.3	22.4
DCP	80.8	72.9	32.7	1.23	37.6	22.8
DBP	78.3	70.5	34.8	1.31	37.7	23.5
SH	77.5	70.5	33.6	1.28	38.1	22.8
Oliveira et al. [42]						
MSMD	80.9	74.9	27.3	1.1	40.4	19.9
MSHD	80.3	74.0	25.3	0.99	39.1	18.1
HSMD	82.6	77.0	26.4	0.88	33.3	18.6
HSHD	82.3	76.7	26.9	0.93	34.6	19.1
Santos-Silva et al. [43]						
LA	79.0	71.5	30.9	1.35	43.8	21.9
MA	74.6	66.8	30.9	1.47	47.6	20.5
HA	70.2	62.1	30.4	1.53	50.3	18.7

^1^ CON: control. DBP: dehydrated beet sugar pulp. DCP: dehydrated citrus pulp. HA: high alfalfa. HSHD: high-starch, high-degradability. HSMD: high-starch, mid rumen degradability. LA: low alfalfa. MA: medium alfalfa. MSHD: mid-starch, high rumen degradability. MSMD: mid-starch, mid rumen degradability. SH: soybean hulls. ^2^ Calculated with Equation (4) of Sauvant et al. [28].

**Table 5:** Intramuscular FA contents and calculated methane production (CH_4_, g/d) of lambs allocated to several experimental treatments in the studies of Costa et al. [41], Oliveira et al. [42] and Santos-Silva et al. [43]. Data expressed as g per 100 g of total fatty acids.

Treatments ^1^	C17:0 *iso*	C17:0 *anteiso*	*Cis*-11 18:1	*Trans*-11 18:1	CH_4_^2^
Costa et al. [41]					
CON	0.20	0.30	1.41	0.91	21.1
DCP	0.17	0.23	1.27	1.77	19.5
DBP	0.18	0.33	1.39	1.62	21.3
SH	0.27	0.32	1.29	3.56	15.3
Oliveira et al. [42]					
MSMD	0.19	0.31	1.33	0.69	17.9
MSHD	0.20	0.34	1.29	0.72	16.6
HSMD	0.15	0.38	1.42	0.58	18.5
HSHD	0.21	0.35	1.34	0.66	17.6
Santos-Silva et al. [43]					
LA	0.19	0.30	1.18	0.91	20.0
MA	0.19	0.27	1.06	1.67	18.7
HA	0.22	0.30	1.11	2.32	16.9

^1^ CON: control. DBP: dehydrated beet sugar pulp. DCP: dehydrated citrus pulp. HA: high alfalfa. HSHD: high-starch, high-degradability. HSMD: high-starch, mid rumen degradability. LA: low alfalfa. MA: medium alfalfa. MSHD: mid-starch, high rumen degradability. MSMD: mid-starch, mid rumen degradability. SH: soybean hulls. ^2^ Calculated with Equation (3) of the present study and scaled to average body weight (i.e., calculated value was multiplied by the ratio average body weight in the study/19.8).

## Data Availability

The data presented in this study are available in the article.
